# Outpatient Management of COVID-19 Disease: A Holistic Patient-Centered Proposal Based on the Greek Experience

**DOI:** 10.3390/jpm11080709

**Published:** 2021-07-23

**Authors:** Adamantia Liapikou, Eleni Tzortzaki, Georgios Hillas, Miltiadis Markatos, Ilias C. Papanikolaou, Konstantinos Kostikas

**Affiliations:** 1 6th Respiratory Department, Sotiria Chest Diseases Hospital, 11527 Athens, Greece; 2 Respiratory Outpatient Clinic, Heraklion, 71305 Crete, Greece; tzortzaki.elena@gmail.com (E.T.); markatosmiltiadis@gmail.com (M.M.); 3 5th Respiratory Department, Sotiria Chest Diseases Hospital, 11527 Athens, Greece; ghillas70@yahoo.gr; 4 Pulmonary Department, Sarcoidosis Clinic, General Hospital of Corfu, 49100 Corfu, Greece; icpapanikolaou@hotmail.com; 5 Respiratory Medicine Department, University Hospital of Ioannina, 45500 Ioannina, Greece; ktkostikas@gmail.com

**Keywords:** COVID-19, outpatient, management, Greek, proposal

## Abstract

Novel coronavirus disease 2019 (COVID-19) is an infectious disease caused by the severe acute respiratory syndrome coronavirus 2 (SARS-CoV-2), has become a worldwide pandemic and affected more than 227 countries or territories, resulting in more than 179 million cases with over 3.890.00 deaths, as of June 25, 2021. The Hellenic Thoracic Society (HTS) during the second wave of COVID-19 pandemic released a guidance document for the management of patients with COVID-19 in the community and in hospital setting. In this review, with guidance the HTS document, we are discussing the outpatient management of COVID-19 patients, including the preventive measures, the patients’ isolation and quarantine criteria of close contacts, the severity and risk stratification, including the decisions for advanced hospitalization, and the disease management at home in patients with mild disease and after hospital discharge for those with more severe disease.

## 1. Introduction

Novel coronavirus disease 2019 (COVID-19) is an infectious disease caused by the newly discovered severe acute respiratory syndrome coronavirus 2 (SARS-CoV-2) that was declared a Public Health Emergency of International Concern by the World Health Organization (WHO) on 30 January 2020 [1,2]. People infected with the COVID-19 virus are most likely to experience only mild symptoms and recover without specialized treatment [2,3]. However, some people will experience more severe disease leading to hospitalization, and very few will develop critical disease requiring ICU admission. In published studies mainly from China and USA, ICU admission ranged from 7 to 14%, invasive mechanical ventilation was used in 29% to 75% of these critically ill patients, and mortality of ventilated patients was extremely variable, ranging from 12% to 81% [4,5].

Internationally, healthcare services delivery is being compromised due to the surge in the number of infected patients during this COVID-19 pandemic [1]. Because the spectrum of forms of the disease varies from asymptomatic to severe, public health systems have been mobilized at all levels. In Greece, the total number of cases was 419,909, while since the beginning of the epidemic, a total of 12,613 deaths have been recorded until 24 June 2021 [6]. The Hellenic Thoracic Society (HTS) during the second wave of COVID-19 pandemic released a guidance document for the management of patients with COVID-19 in the community and in hospital setting [7]. The HTS proposal for COVID-19 was in line with the EMA guidance [8], but a little different from USA (IDSA) guidelines regarding the acceptance of treatment with neutralizing antibodies (bamlanivimab or combination of casirivimab/imdevimab or bamlanivimab/etesevimab) for outpatients patients with COVID-19 [9].

Greece has been one of the countries with relatively good performance in the pandemic so far, despite the significant gaps in the healthcare system, mainly due to the efforts of physicians and allied healthcare professionals, both in the public hospital system and in the primary care/private sector [10]. During the first wave of the pandemic, there was a close collaboration among primary care physicians, secondary facilities, designated hospitals, and official authorities in the management of patients with COVID-19 that was important in the management of the initial crisis at the time [11].

The main reason that the management of the first wave of COVID-19 pandemic achieved relatively good results in Greece was the quick decision of the Greek government to launch early social distancing measures, resulting in limiting the burden of the COVID-19 outbreak and prevention of the healthcare system becoming overwhelmed.

However, the second and third waves of the COVID-19 pandemic generated negative records in Greece regarding the number of hospitalized patients in COVID-19 clinics and in ICUs and the number of related deaths. Thus, the holistic management of the pandemic (outpatient management, home management, and post-discharge management) acquired a fundamental role.

In this review, we discuss the outpatient management of COVID-19 patients, including the preventive measures; the patients’ isolation and quarantine criteria of close contacts; the severity and risk stratification, including the decisions for advanced care (hospitalization); and the disease management at home in patients with mild disease and after hospital discharge for those with more severe disease.

## 2. Preventive Measures

Physical distancing along with facial covering, hand hygiene, and adequate ventilation are crucial measures to reduce the spread of SARS-CoV-2 by stopping chains of transmission and preventing new ones from appearing [12,13,14,15,16]. A recent systematic review of 172 studies, including 25,697 patients on COVID-19, SARS, and MERS, provides strong evidence that current policies of at least 1 m physical distancing are associated with a large reduction in infection but distances of up to 2 m might be more effective [17]. Paramount available evidence suggests that face masks protect people by reducing the risk of infection when they cough, sneeze, or speak, especially when caring for someone with COVID-19 [12,13,14,15,16,17]. The optimum use of face masks, in the community, could depend on various factors thus strict instructions should be followed when putting on, wearing and removing the mask. Besides, the level of protection is decreases, the longer the mask is worn [12,13,17]. 

There are three main types of facial masks: *A. Non-medical fabric masks* that can be used by the general public under the age of 60 without an underlying health conditions; these masks, when reusable should be washed regularly. B. *Medical masks* that are made from a minimum of three layers of synthetic nonwoven material and offer high protection. Medical masks are used by both health-care workers and the general public, as well as by patients with underlying health conditions, by people over the age of 60 years, and by anyone with a suspected or confirmed COVID-19 infection. *C. Respirators*, such as FFP2 (N95) or FFP3 (N99), which provide maximum protection and are designed precisely for certain groups, such as healthcare professionals who are working in direct contact with COVID-19 patients. They should be fitted to the person using them and are not recommended for use by others [10,11]. In the recent months of widespread availability of COVID-19 vaccines, the CDC recommended that fully vaccinated people can continue activities without wearing a mask in several situations when outside, such as small outdoor gatherings with family and friends, outdoors dinning at restaurants, or outdoor exercise; however, fully vaccinated individuals should continue to wear a face mask at home when caring for a COVID-19 patient, in indoor public spaces and outdoors where there is a high risk of COVID-19 transmission, such as at a crowded event, or in all forms of public transportation [18].

In addition, *handwashing* with soap and water for at least 20 s or the use of at least 60% alcohol-based hand sanitizers can prevent the spread of COVID-19 infection [19]. Hand cleaning must be done before and after touching eyes, nose, or mouth or the face mask; when entering and leaving a public place; or when touching an item or surface that may be frequently touched by others, such as door handles, tables, shopping carts, and screens [19].

Last, SARS-CoV-2 viral particles spread between people more readily indoors than outdoors. Thus, ensuring proper *ventilation* with outdoor fresh air can reduce the airborne concentrations of viral particles and, thus, reduce the overall viral dose to occupants. The CDC recommends increasing ventilation with outdoor air and air filtration as important components of a larger strategy that includes social distancing, face coverings, surface cleaning, and handwashing to reduce viral exposure [20].

## 3. Patients’ Isolation and Quarantine Criteria of Close Contacts 

The isolation and quarantine criteria depend on the severity of the contact, the vaccination status and/or the previous COVID-19 disease and its severity. Some specific recommendations are the following.

Close contacts of a confirmed COVID-19 case are the people whose contact with the case is detected up to 48 h before the onset of its symptoms or its laboratory diagnosis. Close contacts should be quarantined at home or in another area which they will indicate as a quarantine area, on their own, for at least 14 days from their last contact with the confirmed case [21].

Fully vaccinated individuals or those with a confirmed SARS-CoV-2 infection in the past 6 months, who have been at high risk for exposure to a COVID-19 patient but remain asymptomatic do not need to be quarantined as the risk of infection is very low. These individuals should be self-monitored (daily temperature measurement, vigilance for symptoms) for 14 days after exposure and adhere to social distance measures.

The criteria taken into account in COVID-19 patients regarding the isolation time are the following [22]: (1) clinical improvement of symptoms, (2) time from the onset of symptoms, (3) severity of the disease, (4) patient’s immune status, and (5) indication of clearance of the viral RNA from the secretions of the upper respiratory system.

Asymptomatic individuals who tested positive for SARS-CoV-2 should be isolated for 10 days from the date of sampling and laboratory diagnosis.

Patients with mild disease. The main criterion for removing the isolation in these cases is the complete remission of fever and the improvement of clinical symptoms, mainly from the respiratory system. The completion of 10 days of isolation and 3 days of apyrexia and remission of other clinical symptoms, other than fever, greatly reduces the likelihood of transmission of the virus [23].

Immunocompetent patients with serious illness end their isolation after at least 14 to 20 days tested positive for SARS-CoV-2 and 72 h of complete remission of fever without taking antipyretic and improvement of their symptoms from the respiratory system. Alternatively, they end their isolation after at least 14 to 20 days tested positive for SARS-CoV-2 and two consecutive negative molecular detection tests of the virus in secretions of the respiratory tract with a difference of 24 h sampling.

Immunocompromised patients with serious illness end their isolation after 20 days tested positive for SARS-CoV-2 and 72 h of complete remission of fever without taking antipyretic and improvement of their symptoms from the respiratory system. Alternatively, they end their isolation after 20 days tested positive for SARS-CoV-2 and two consecutive negative molecular detection tests of the virus in secretions of the respiratory tract with a difference of 24 h sampling [24].

The return of a positive case to work should take into account the presence of immunosuppression, the frequency of contact of the case with susceptible individuals with severe COVID-19 disease, and whether the affected individual’s work is associated with areas characterized by high risk of transmission and confluence. (e.g., closed structures, chronically ill units, prisons or immigrant/refugee accommodation facilities).

## 4. Risk Stratification of COVID-19 Disease in Outpatients

Once a person had laboratory confirmation of COVID-19 infection, irrespective of clinical signs and symptoms, it is a Confirmed Case and needs to be managed according to the severity of the disease.

### 4.1. Clinical Spectrum

Generally, outpatient management is appropriate for most patients with COVID-19; in approximately 80% of patients, illness is mild and does not warrant medical intervention or hospitalization; however, the elderly and patients with comorbidities tend to have more severe disease. The clinical spectrum of the disease ranges from asymptomatic to mild symptoms such as cough, fever, and myalgias to pneumonia to acute respiratory distress syndrome, and sepsis with septic shock to multiorgan failure [25]. Less common symptoms are sputum production, headache, sore throat, hemoptysis, rhinorrhea and even gastrointestinal symptoms, such as nausea and diarrhea. Anosmia (loss of smell) is being reported in some cohorts as a presenting symptom occurring even before any other clinical feature.

### 4.2. Clinic Evaluation

For patients evaluated in an outpatient clinic (if feasible, a respiratory/COVID-19 clinic), we assess the patient’s respiratory and circulatory status, and we evaluate for other potentially treatable causes of symptoms. Based on a careful clinical history and physical exam, including vital signs as well as measurements of oxygen saturation at rest and with ambulation, we then determine if the patient is appropriate for self-care, outpatient therapy (if available through a clinical trial), or transfer to the ED for further evaluation or possible inpatient hospital admission. Important issues are listed below.

- Infection control: for all patients, we reinforce the importance of infection control and self-isolation and provide instructions on the anticipated duration of isolation.
- Remote (telehealth) management is preferred for the majority of patients for the following reasons: (a) Remote management can prevent unnecessary in-person medical visits, including visits to urgent care facilities and Eds. (b) In-person healthcare provider visits require the patient to leave their home, traveling via public, private, or emergency transport and potentially exposing others to SARS-CoV-2. (c) Upon arrival at a health care facility, patients may expose other patients and health care workers to the virus [26,27,28].
- The use of home pulse oximetry monitoring is recommended in patients seen in the ambulatory or ED setting and discharged home, but it is not always a flawless solution [26]. However, and as dyspnea may not correlate with the presence or degree of hypoxia in all patients, along with risk factors for developing severe disease (Table 1), pulse oximetry can be used to guide clinicians in determining whether a patient requires in-person evaluation [29].

Disease can be categorized into mild, moderate, severe, and critically ill for appropriate management. 

Stratification by predicted risk, most commonly for death, can support clinical judgement and potentially assist clinicians in community settings to decide how urgently to refer patients to hospital. Not all patients infected with SARS-CoV-2 will require hospitalization, but even among those who initially experience mild symptoms, a sizeable proportion remain at risk of subsequent life-threatening of clinical decline.

### 4.3. Risk Stratification

The patient-centered continuum of care management approach is based on the stratification by risk for developing severe disease and the close monitoring for respiratory decompensation. The intensity (frequency and duration) of outpatient follow-up will depend on the individual patient’s risk for development of severe disease (Table 1) and may vary by institution, region, and resource availability. COVID-19 has also disproportionately affected residents of nursing homes and long-term care facilities, due to the high proportion of frail older adults and those with underlying chronic conditions [30]. These factors increase both the prevalence and severity of infection, resulting in high mortality rates among this population. Specifically, in addition to increasing age, established risk factors for severe disease include specific comorbidities, obesity, and smoking habit [31,32,33].

Therefore, the proposal of the Hellenic Thoracic Society for the management of COVID-19 at home is a combination of clinical presentation and comorbidities (Table 2).

Symptoms showing more severe disease are as follows:

- severe shortness of breath at rest or difficulty breathing
- reduced oxygen saturation levels measured by pulse oximetry
- coughing up blood
- collapse or fainting (syncope)
- new confusion

In detail, the risk of severity is classified as follows: (1) Mild, if the patient is asymptomatic or with mild symptoms and the proposed management is home care. (2) Moderate, if a patient with comorbidities or >65 years old has fever < 38.5 °C, but no tachypnea and without hypoxemia (SaO_2_ > 94%) and he/she needs frequent remote management. (3) Severe with symptoms and hypoxemia or dyspnea, he/she should be transferred to the ED.

## 5. Management of Patients with COVID-19 at Home

The outpatient management of patients with COVID-19 consists of the below components: the role of specific therapies, the administration of antibiotics, and anti-coagulation issues (Table 3).

### 5.1. Specific COVID-19 Therapies under Investigation

#### 5.1.1. Chloroquine, Hydroxychloroquine, Azithromycin

Based on possible in vitro antiviral activity against SARS-CoV-2, antimalaria drugs chloroquine and hydroxychloroquine were used in clinical practice at the onset of the pandemic in 2020 [37]. Randomized trials examined the effect of hydroxychloroquine (400 mg bid) with or without azithromycin (500 mg/d) in hospitalized patients with mild or moderate COVID-19 pneumonia and found no benefit on clinical and viral clearance outcomes [38,39]. Therefore, their administration in an outpatient basis is not recommended. Hydroxychloroquine has also proven ineffective as post-exposure prophylaxis in high-risk contacts to prevent from infection and COVID-19 illness, in two double-blind randomized trials [34,40]. Meta-analysis including 28 trials, with WHO-Solidarity and Recovery trials among them, found no benefit and increased mortality for both hydroxychloroquine and chloroquine (odds ratio 1.11, 95% CI: 1.02, 1.20; odds ratio 1.77, 95% CI: 0.15, 21.13, respectively) [35].

#### 5.1.2. Colchicine

Treatment with colchicine has been examined against COVID-19 due to its anti-inflammatory and cardioprotective properties, both in hospitalized and out-patient settings, in Greece as well [36]. COLCORONA was a multicenter, double-blind, randomized phase 3 study, in which 4488 outpatients with recent (≤24 h) diagnosis of COVID-19 received either colchicine 0.5 mg twice a day for 3 days and then 0.5 mg once a day for 27 days or placebo. The study included cases with solely clinical diagnosis and with polymerase chain reaction (PCR)-based diagnosis. Eligible were patients of at least 40 years of age with comorbidities and/or fever above 38.4 °C and/or hematological cytopenias. The primary endpoint of death or hospital admission was not met for the whole study population but was met in the PCR-confirmed COVID-19 subpopulation (OR 0.75, 0.57–0.99; *p* = 0.042). Regarding secondary endpoints, serious adverse events were reported equally among the colchicine and placebo group, pneumonia was more frequent in the placebo group and diarrhea was much more common in the colchicine group [52]. Positive results are shown in smaller retrospective studies in terms of time to recovery, serious adverse events, and biomarkers such as neutrophil to lymphocyte ratio [53].

#### 5.1.3. Ivermectin

Ivermectin is an old anti-parasitic drug repurposed against COVID-19 that was extensively studied in mild, moderate, and severe COVID-19. In a small prospective placebo-controlled trial where 24 non-hospitalized patients with mild disease and no risk factors for severe disease were randomized to take ivermectin 400 mcg/kg (single dose) or placebo within 3 days of disease onset, ivermectin was not associated with increased PCR negativity at day 7 [42]. In a larger RCT of 400 mild COVID-19 outpatients with time to resolution of symptoms as primary endpoint, a 5-day course of ivermectin at a dose of 300 μg/kg/day failed to demonstrate any benefit versus placebo [41].

#### 5.1.4. Inhaled Budesonide

In one randomized controlled phase 2 trial, investigators administered high dose inhaled budesonide (1600 mcg/day) for a mean time of 7 days, compared to standard of care, in early, mild non-hospitalized COVID-19 patients. The rationale was the underrepresentation of chronic obstructive pulmonary diseases among severe COVID-19. The budesonide arm met the primary endpoint, as it led to 91% relative reduction in clinical deterioration (emergency care visits and hospitalizations) in both the intention to treat and the per protocol populations, with a number needed to treat of eight patients needed to be treated with budesonide to avoid 1 deterioration. Budesonide also shortened self-assessed clinical recovery and other patient-reported outcomes by 1 day but had no effect on oxygen saturation and viral load accelerated clearance. Albeit statistically significant, primary end-point incidents in the per-protocol population were few (11/70 participants) and of questionable clinical significance. Moreover, the young age of participants (mean age 44 years old) and the presence of 1 or less comorbidities reduce the generalizability of the efficacy of this intervention, until further studies in at-risk for clinical deterioration patients take place [54].

#### 5.1.5. Fluvoxamine

Fluvoxamine is a selective serotonin reuptake inhibitor that has been tested against COVID-19 as a potential cytokine production regulator. In one small, randomized trial in early COVID-19 patients not requiring hospitalization, fluvoxamine reduced adverse outcomes (need for oxygen, desaturation below 92%, shortness of breath) by 8.7% (95% CI, 1.8–16.4%; log-rank *p* = 0.009) [44]. Due to the small sample size and the absence of confirmation in larger studies with more robust outcomes, the role of fluvoxamine remains uncertain.

#### 5.1.6. Monoclonal Antibodies

Monoclonal antibodies are generally administered in patients with mild or moderated disease with risk factors for severe disease (age, comorbidities, and high BMI). Although they are given intravenously in-hospital, they are purposed for patients not needing hospitalization. The FDA has issued emergency use authorization, but still issues remain regarding their use and efficacy. Bamlanivimab and etesevimab are potent anti-spike monoclonal antibodies developed from the plasma of recovered patients. In the interim analysis of a phase 2 clinical study of bamlanivimab alone in three different doses, Chen et al. reported a significant 3.4 times reduction of the viral load at day 11, when investigational drug dose was 2800 mg. Further, hospitalizations were observed in 1.6% (5 of 309 patients) in the bamlanivimab group and 6.3% (9 of 143 patients) in the placebo group. These rates were 4% versus 15% in the post hoc analysis of patients with age ≥ 65 and BMI ≥ 35 [48]. In the concluding phase 2/3 study examining bamlanivimab alone or in combination with etesevimab in a single infusion, only the combination (at a dose of 2800 mg each) led to a −0.57 decrease in log viral load at day 11 compared to placebo (between-group difference, −0.57; 95% CI, −1.00 to −0.14, *p* = 0.01). Among the various secondary endpoints met by the combination arm, the study reports a relative decrease in hospitalizations and emergency department visits (−4.9%; 95% CI, −8.9% to −0.8%; *p* = 0.049). The study included early symptomatic patients, 67% among them having at least one risk factor for severe disease (age ≥ 55 or BMI ≥ 30 or ≥1 comorbidity) [55]. Another anti-spike neutralizing antibody combination (REGN-COV2, containing casirivimab and imdevimab), studied in a similar population, was found to accelerate viral clearance by day 7 and improve clinical outcomes, in patients with high initial viral load and negative serum anti-SARS-CoV-2 antibodies, without safety issues [56]. Matters to be resolved regarding their use are logistics issues, dosing, and resistance. Administration in Hospital environment is challenging in present times where patients should be isolated and healthcare systems are overloaded. Dosing approved in United States for bamlanivimab/etesevimab is 700 mg/1400 mg, based on unpublished data [57]. Last, concerns arise due to evidence that certain mutated variants of SARS-CoV-2 exhibit resistance in vitro mainly to bamlanivimab and less to REGN-COV2 constituents [45,49].

#### 5.1.7. Other Agents

In ambulatory patients with mild to moderate COVID-19, administration of high dose zinc, ascorbic acid, or both failed to decrease the duration of symptoms compared to standard of care [43]. Lopinavir/ritonavir when administered in an outpatient setting failed to demonstrate any benefit in hospitalizations or expedited viral clearance [50]. Peginterferon lambda is a type III interferon involved in innate immunity against respiratory pathogens. This molecule was administered once subcutaneously in early ambulatory COVID-19 patients in a double-blind randomized placebo-controlled trial. Patients treated with interferon were more likely to have undetectable virus by day 7 (odds ratio 4.12, 95% CI 1.15–16.73, *p* = 0.029). No significant adverse events were reported [51]. Finally, the RECOVERY trial that exhibited a benefit of dexamethasone in hospitalized patients on oxygen supplementation, did not show any benefit in patients who do not require respiratory support; therefore, systemic corticosteroids are not indicated for out-patients, unless it is provided in the context of asthma or chronic obstructive pulmonary disease exacerbations or other acute conditions [58].

### 5.2. The role of Antibiotics

PRINCIPLE was the first study to access effectiveness of antibiotics, specifically azithromycin, as a standalone treatment in the community. Two-thousand-two-hundred-and-sixty-five high-risk outpatients (aged > 65 years or > 50 years plus comorbidities) were randomized; among them, 540 to azithromycin 500 mg/day for 3 days and the rest to usual care or other interventions. The azithromycin group did not present faster recovery nor reduced hospitalizations compared to usual care. Therefore, its use should be avoided in the outpatient setting, as it additionally confers to antibiotic resistance and may cause delays in seeking more appropriate therapies [46].

### 5.3. The Role of Anticoagulation

Patients with known cardiovascular diseases or risk factors for vascular disease (age, smoking, diabetes, hypertension) are well known to be at risk for severe and critical COVID-19. COVID-19 is well known to promote hypercoagulation and bleeding via endothelial damage. The VAS-European Independent Foundation in Angiology/Vascular Medicine issued guidelines recommending that these patients, upon diagnosis of COVID-19, should be at close monitoring at the community, through a primary health network and e-health tools. A prophylactic dose of LMWH, rivaroxaban 10 mg, or betrixaban 80 mg once daily could be considered for thromboprophylaxis in high VTE (venous thromboembolism) risk COVID-19 outpatients. High risk, according to this statement, may be estimated via IMPROVE and PADUA scores which include parameters such as obesity, older age, previous VTE, malignancy, thrombophilia, and acute illness/immobilization the past 7 days [47]. Various regimens for VTE prophylaxis in outpatients at risk with COVID-19 are currently under investigation in many clinical trials [59,60]. Another retrospective study including 715 out-patients, reported no major cardiovascular or thromboembolic event in this population [61]. For outpatient COVID 19 patients with proximal deep vein thrombosis or pulmonary embolism and no drug–drug interactions, CHEST panel report recommends apixaban, dabigatran, and rivaroxaban. or edoxaban. Initial parenteral anticoagulation is needed before dabigatran and edoxaban. For patients who are not treated with a DOAC, the panel suggests vitamin K antagonists over LMWH (for patient convenience and comfort). Parenteral anticoagulation needs to be overlapped with vitamin K antagonists [62].

## 6. Management of Patients with COVID-19 after Hospital Discharge

### 6.1. Isolation Management and Health Monitoring

After hospital discharge, patients are recommended to continue 14 days of isolation management and health monitoring, wear a mask, live in a single room with good ventilation, avoid close contact with family members, eat separately, keep hands clean, and avoid outdoor activities. Discharged patients should have follow up visits after 4–6 weeks from discharge, and an evaluation ideally from a multidisciplinary team 12 weeks post-discharge [63]. When a period of overall isolation of 14–21 days has been completed, no negative Polymerase Chain Reaction (PCR) test for SARS-CoV-2 is needed [64].

### 6.2. Venous Thromboembolism (VTE) Prophylaxis

Current evidence shows that severe COVID-19 can be complicated by coagulopathy. In the most critically ill patients, this manifests as disseminated intravascular coagulation (DIC), which is a pro-thrombotic condition with a high risk of Venous Thromboembolism (VTE). In acutely ill hospitalized patients with COVID-19 (in the absence of a contraindication), anticoagulant thromboprophylaxis over no anticoagulant thromboprophylaxis is generally recommended [65]. The Italian Society on Thrombosis and Haemostasis recommends prophylaxis throughout hospitalization and for an additional 7 to 10 days post discharge [66]. The American Society of Hematology recommends against extended duration prophylaxis in hospitalized medical patients [67]. The CHEST Expert Panel Report recommend inpatient thromboprophylaxis only over inpatient plus extended thromboprophylaxis after hospital discharge in patients with COVID-19. However, extended thromboprophylaxis in patients with COVID-19 at low risk of bleeding should be considered if emerging data on the post-discharge risk of VTE and bleeding indicate a net benefit of such prophylaxis [65].

For certain patients at high risk of VTE without COVID-19, post-discharge prophylaxis has been shown to be beneficial [68]. The Food and Drug Administration approved the use of rivaroxaban 10 mg daily for 31 to 39 days in these patients. Inclusion criteria for trials that studied post-discharge VTE prophylaxis included [69,70] a modified International Medical Prevention Registry on Venous Thromboembolism (IMPROVE) VTE risk score ≥ 4; or a modified IMPROVE VTE risk score ≥ 2 and D-dimer level > 2 times the upper limit of normal. Any decision to use post-discharge VTE prophylaxis for patients with COVID-19 should include the consideration of individual patients’ risk factors for VTE, including reduced mobility, bleeding risks, and feasibility [70].

### 6.3. Deep Venous Thromboembolism (DVT) or Pulmonary Embolism (PE)

In critically ill COVID-19 patients with proximal DVT or PE, the parenteral over oral anticoagulant therapy is recommended. In critically ill COVID-19 patients with proximal DVT or PE who are treated with parenteral anticoagulation, low-molecular-weight heparin (LMWH) or fondaparinux over unfractionated heparin (UFH) is recommended. For COVID-19 patients with proximal DVT or PE, anticoagulation therapy for a minimum duration of three months is generally recommended [66].

### 6.4. Post-COVID-19 Syndrome

Some hospitalized COVID-19 patients develop post-COVID-19 syndrome (long COVID). This syndrome is also known as post-acute COVID-19 syndrome, chronic COVID, long-haul COVID, post-acute sequelae of SARS-CoV-2 infection (PASC), and post-COVID conditions [71,72]. The post-COVID-19 syndrome is characterized by signs and symptoms that develop during or after an infection consistent with COVID-19, continue for more than 12 weeks, and are not explained by an alternative diagnosis. Ongoing symptomatic COVID-19 is defined as signs and symptoms from 4 weeks up to 12 weeks. The syndrome is not thought to be linked to disease severity or specific signs and symptoms during the acute phase of illness [71,72].

There is no standardized case definition and case definitions vary. The US Centers for Disease Control and Prevention defines post-COVID conditions as an umbrella term for the wide range of health consequences that are present more than 4 weeks after infection with SARS-CoV-2. Protracted symptoms are common in many viral and bacterial infections. Neurological symptoms are similar to symptoms of other neurological conditions such as chronic fatigue syndrome and functional neurological disorder [73].

A holistic, person-centered approach that includes a comprehensive clinical history and appropriate examination that involves assessing physical, cognitive, psychological, and psychiatric symptoms, as well as functional abilities is strongly recommended. The referral of such patients to the relevant specific medical services is encouraged.

### 6.5. Physical Therapy (PT)

Prolonged hospital admission or isolation greatly reduced the amount of exercise in this stage, resulting in muscle weakness, fatigue and low exercise endurance, and weakness. The purpose of PT in discharged patients with COVID-19 is to enable patients to return to society, restore their organic functions, and prevent psychological disorders. PT at home mainly uses remote guidance, psychological support, social education, and other means to let patients understand the importance of PT, through brochures or videos to make patients understand respiratory rehabilitation, adopt a healthy lifestyle, and to promote their return to their families and society.

Therapeutical interventions proposed in this phase comprised of the following: (1) Aerobic exercise. The intervention should last for at least 6 weeks, five times a week, 30–60 min/day, and with an intensity that is increased by 10% every week. (2) Resistance training: Squatting is permitted, and medium weight items can be carried. (3) Balance training: cross obstacles. (4) Breathing training: normal breathing mode is used for breathing training; abdominal breathing; pursed-lip breathing; thoracic expansion exercise are other exercises suggested [74].

### 6.6. Rehabilitation

Following discharge of severe COVID-19 cases and/or COPD exacerbation, rehabilitation professionals can provide graded exercise, education on energy conservation and behavior modification, home modification, and assistive products, as well as rehabilitation for any specific individual impairments. During the long-term recovery of severe COVID-19, patients may benefit from pulmonary rehabilitative interventions, which target physical and respiratory impairments, and include a combination of graded exercise, education, activity of daily living, and psychosocial support [75].

### 6.7. Reassessment of the Respiratory System

As the COVID-19 pandemic represents a new disease, the long-term pulmonary outcomes in survivors of COVID-19 are unknown. Evidence from other coronavirus pneumonias, such as SARS and Middle East Respiratory Syndrome (MERS), suggests that impaired lung function and parenchymal opacities persist only in a minority of patients not having required mechanical ventilation. However, 3 months after admission for COVID-19, one-fourth of the participants have been reported to have chest CT opacities and reduced diffusing capacity, and their admission to ICU has been associated with pathological CT findings [76]. Additionally, four months after hospitalization for COVID-19, a cohort of patients frequently reported symptoms not previously present, and lung scan abnormalities were common among those who were tested [77].

Following SARS-CoV-2 pneumonitis, a cohort of patients is left with both radiological inflammatory lung disease and persistent physiological and functional deficit. Early treatment with corticosteroids was well tolerated and associated with rapid and significant improvement [78]. Thus, 12 weeks after the discharge of a hospitalized patient, a clinical assessment of respiratory system with a chest *x*-ray or with a chest CT scan is recommended. In patients with persistent symptoms and/or radiological findings, a further assessment with lung function tests (including DLco) could provide clinically meaningful information.

## 7. Conclusions

In this review, we have summarized international recommendations and the Greek experience for the outpatient evaluation and management of patients with COVID-19, both in in patients with mild disease that will not require hospitalization, as well as after discharge for those with more severe disease that have been hospitalized in COVID-19 wards or in the ICU. As a reflection of our health politics on COVID-19 management, although the case fatality rate (CFR) varies widely between countries, CFR of the confirmed cases for Greece was approximately 5% during the last year of the pandemic in comparison to 14–4% of CFR for Italy and 11–2% for Spain [79].

Despite the fact that the management of COVID-19 patients remains an evolving field, our current understanding of the disease and the body of evidence available allows us to provide comprehensive evidence-based care to the majority of our patients. The isolation/quarantine suggestions are likely to change as the vaccination status in certain populations increases and should be closely monitored at a local level. As the pandemic evolves, more effective outpatient treatments (e.g., monoclonal antibody combinations or inhaled treatments with local efficacy on the target organ of the virus, the lung) that can be more easily administered will likely be the winner in the race for early intervention that will reduce the need for hospitalization and ICU admission. The biggest unmet need remains the appropriate care of COVID-19 survivors, especially of those with long-standing symptoms and respiratory or other organ system consequences. This is an area where respiratory physicians and multidisciplinary teams that will address holistically the patients with symptoms and/or disability will play a cardinal role in the minimization of the consequences of this disease.

## Figures and Tables

**Table 1:** Risk factors for severe disease.

Established Risk Factors	Possible Risk Factors
Cancer	Cystic fibrosis
Chronic kidney disease	Thalassemia
Chronic obstructive pulmonary disease	
Down syndrome	
Immunocompromised state from solid organ transplant	
Obesity (body mass index [BMI] ≥ 30 kg/m^2^)	
Serious cardiovascular disease (e.g., heart failure, coronary artery disease, cardiomyopathy)	
Smoking	
Sickle cell disease	
Type 2 diabetes mellitus	
Pregnancy	

**Table 2:** Management of confirmed COVID-19 at home.

Risk Stratification	Characteristics	Proposed Management
Mild	Asymptomatic or mild symptoms	Home care ^1^ If no improvement or deterioration → hospital admission
Moderate	Fever < 38.5 °C, cough, pharyngalgia + Comorbidities ^2^ orAge > 65 years old or CXR or CT (+) Respiratory rate ≤16/min or SaO_2_ > 94%	Clinical and laboratory evaluation at home (complete blood count, CRP) Azithromycin + chloroquine phosphate or hydroxychloroquine ^3^ ± antibiotics for community acquired pneumonia If no improvement or deterioration → hospital admission
Severe	Fever ≥ 38.5 °C, cough, fatigue, dyspnea + Comorbidities or Age > 65 years old + CXR ή CT (+)	Hospital admission Consult: Management algorithm for patients with confirmed COVID-19 in hospital setting

If patient is in respiratory distress or has SaO_2_ ≤ 93%, hospital admission is recommended regardless of risk stratification status. ^1^ Family doctor regularly assesses clinical status and laboratory tests. ^2^ Comorbidities: Chronic lung and/or heart diseases, immunosuppression (cancer patients undertreatment, solid organ or hematopoietic stem cell transplantation, immunodeficiencies, not controlled HIV infection, corticosteroids, or other immunosuppressive drugs), diabetes mellitus, renal failure, liver failure, morbid obesity (ΒΜΙ > 40). ^3^ Azithromycin: 500 mg o.d. for 7 days (be careful for possible cardiotoxicity). Hydroxychloroquine: 400 mg b.i.d. on day 1, following 200 mg b.i.d. for the next 5–7 days (or 400 mg o.d). Chloroquine phosphate: 500 mg b.i.d for 5–7 days (be careful for possible cardiotoxicity: QT prolongation > 500 msec, myasthenia gravis, porphyria, epilepsy, retinal toxicity, G6PD deficiency and drug interactions (consult http://www.COVID19-druginteractions.org, accessed on 29 June 2020. If initial QTc: 450–500, daily ECG and monitoring of blood parameters).

**Table 3:** Outpatient treatment regimen trials against COVID-19.

Positive Results	Negative Results	Pending/Inconclusive Results
Colchicine [34,35,36]	Chloroquine, hydroxychloroquine ± azithromycin [33,37,38,39,40]	Fluvoxamine [41]
Inhaled budesonide [42]	Ivermectin [41,42]	Peginterferon lambda [43]
Bamlanivimab and etesevimab [44]	Zinc plus ascorbic acid [45]	Thromboembolic prophylaxis [46,47]
Casirivimab and imdevimab [48]	Lopinavir plus ritonavir [49]	
	Systemic corticosteroids [50]	
	Azithromycin [51]	

## Data Availability

Not applicable.
